# Genome-wide identification of *MYBL2* in Brassicaceae, with a focus on the expression pattern of regulating anthocyanin synthesis in *Brassica* crops

**DOI:** 10.3389/fpls.2025.1629560

**Published:** 2025-07-01

**Authors:** Chenchen Wang, Qi Zhang, Yi Liu, Wenjie Shen, Shubei Wan, Lunlin Chen, Chen Tan, Daozong Chen

**Affiliations:** ^1^ College of Life Sciences, Ganzhou Key Laboratory of Greenhouse Vegetable, Gannan Normal University, Ganzhou, China; ^2^ Nanchang Branch of National Center of Oilcrops Improvement, Jiangxi Province Key Laboratory of Oil Crops Biology, Crops Research Institute of Jiangxi Academy of Agricultural Sciences, Nanchang, China

**Keywords:** MYBL2, Brassicaceae, anthocyanins, expression pattern, *Brassica* crops

## Abstract

The Brassicaceae family includes the model plant *Arabidopsis thaliana*, along with various vegetables and oil crops, which possess significant economic and scientific value. Notably, three diploid species within the U’s Triangle of *Brassica* have undergone natural hybridization, resulting in the formation of three allotetraploid species, which provides an excellent model for investigating the phylogenetic, evolutionary, and functional differentiation of plant homologous genes. In this study, we systematically identified *MYBL2* homologous genes within the 31 Brassicaceae species, with a total of 48 homologous genes identified from 30 species, and phylogenetic analysis revealed the presence of six subfamilies, Ka/Ks analysis showed that only 10 *MYBL2* homologous gene were positively selected during evolution. We subsequently concentrated on the evolution, gene structure, and conserved domain analysis of 16 *MYBL2* homologous genes across six *Brassica* crops found in U’s Triangle. Our findings indicated that these 16 *MYBL2* homologous genes predominantly clustered into two branches and exhibited a high degree of evolutionary conservation. Further RNA-seq analysis of various tissues and organs from *Brassica* crops demonstrated that *MYBL2* homologous genes were significantly up-regulated in tissues with anthocyanin accumulation. Concurrently, we employed Weighted Gene Co-expression Network Analysis (WGCNA) to identify hub genes regulated by anthocyanin in different tissues of *B. napus*, revealing that *BnaMYBL2.C06* exhibited a strong repressor with anthocyanin biosynthetic genes (ABGs) in petals. Finally, quantitative reverse transcription PCR (qRT-PCR) analysis of *B. napus* leaves, stems, and petals indicated that four *MYBL2* homologous genes were significantly up-regulated in leaves and petals, with expression patterns consistent with those of ABGs. Our results contribute new insights into the transcriptional regulatory mechanisms of anthocyanin in *Brassica* crops.

## Introduction

The Brassicaceae family holds significant scientific and economic value, encompassing various vegetables (e.g., cabbage), ornamental flowers (e.g., violet), oil crops (e.g., rapeseed), and the model plant *Arabidopsis thaliana*. This family serves as an ideal genetic tool and resource for the study of flowering plants. In recent years, researchers have deciphered nearly 90 versions of reference genomes from 31 species, contributing to genetic and evolutionary studies of Brassicaceae species. This work provides a foundation for exploring the phylogenetic relationships and biological functions of cruciferous species (Liu et al., 2022). The genus *Brassica* is particularly important within the family, comprising six species known as U’s triangle: *B. rapa* (AA, 2n = 20), *B. oleracea* (CC, 2n = 18), *B. nigra* (BB, 2n = 16), *B. juncea* (AABB, 2n = 36), *B. napus* (AACC, 2n = 38), and *B. carinata* (BBBCC, 2n = 34) (Warwick, 2011). Notably, three diploid species, *B. rapa*, *B. oleracea*, and *B. nigra*, have experienced genome-wide triploid events during their evolution. Additionally, three allotetraploid species, *B. juncea*, *B. napus*, and *B. carinata*, formed through natural doubling following hybridization, have undergone significant alterations in the number of homologous genes due to chromosomal rearrangements. These species present an ideal model for studying the evolution and function of homologous genes (He et al., 2021). Currently, the chromosome levels of the six *Brassica* species have been published, laying a foundation for the systematic identification and exploration of gene phylogeny, evolution, and functional differentiation (Cai et al., 2020; Perumal et al., 2020; Kang et al., 2021; Song et al., 2021; Tang et al., 2021; Zhang et al., 2023).

Anthocyanins are a significant class of secondary metabolites in plants, responsible for imparting various colors, including red, purple, and blue, to different plant organs. They are widely distributed across petals, leaves, fruits, seeds, and other tissues (Tanaka et al., 2008; Zhao et al., 2022). In plants, the accumulation of anthocyanins aids in pollination and seed dispersal, enhances resistance to pests and diseases, and improves tolerance to abiotic stresses (Shang et al., 2011). Furthermore, anthocyanins act as natural antioxidants, contributing to human health by enhancing immunity, improving vision, delaying aging, preventing chronic diseases, and exhibiting antibacterial, anti-inflammatory, and anti-tumor activities (Zhang and Jing, 2022). The biosynthetic pathway of anthocyanins has been extensively studied in various plants, with most of the genes involved identified. The structural genes encoding anthocyanin synthase are categorized into early biosynthetic genes (EBGs), which primarily include *PAL*, *C4H*, *4CL*, *CHS*, *CHI*, *F3’H*, and *F3H*, and late biosynthetic genes (LBGs), which mainly comprise *DFR*, *ANS*(*LDOX*), *UGT*, and *GST* (Tanaka et al., 2008). The MYB-bHLH-WD40 (MBW) complex, formed by the MYB transcription factor, bHLH transcription factor, and WD40 repeat protein, collaboratively regulates the expression of late structural genes in anthocyanin biosynthesis (Zhao et al., 2013; Khusnutdinov et al., 2022).

Current studies have demonstrated that R2R3-MYB transcription factors, including *PAP1/MYB75*, *PAP2/MYB90*, *MYB113*, and *MYB114*, along with bHLH transcription factors such as *GL3*, *EGL3*, and *TT8*, as well as the WD40 protein *TTG1*, positively regulate anthocyanin biosynthesis in *A. thaliana* (Zhang et al., 2003; Zhao et al., 2013; Naing and Kim, 2018). The main R2R3 MYB regulatory factors, *PAP1/2* and *MYB113/114*, which regulate anthocyanin synthesis in plants, have been reported to enhance anthocyanin production in several species, including maize (Cone et al., 1986), Arabidopsis (Teng et al., 2005), and tomato (Zuluaga et al., 2008). Conversely, negative regulators of anthocyanin synthesis also exist in plants. In *A. thaliana*, the R3-MYB transcription factor *AtMYBL2* inhibits the formation of the MBW complex by interacting with the bHLH transcription factor *TT8* (Matsui et al., 2008). This complex is capable of binding to the *DFR* promoter, thereby repressing the transcription of both *DFR* and *TT8*, which in turn inhibits anthocyanin biosynthesis (Matsui et al., 2008). Following the knockout of the *AtMYBL2* gene in *A. thaliana*, it was observed that the expression levels of the key gene *DFR* and the regulatory gene *TT8* were upregulated, leading to a significant increase in anthocyanin content (Dubos et al., 2008). Furthermore, it was discovered that *HY5* positively regulates anthocyanin accumulation in Arabidopsis by activating *MYBD*, which in turn inhibits *MYBL2* expression (Nguyen et al., 2015). In Zicaitai (*B. rapa*) and Chinese cabbage, the MYB transcription factor *BrMYBL2.1* is regarded as a negative regulator of anthocyanin biosynthesis (Zhang et al., 2020; Kim et al., 2022). In *B. oleracea*, higher anthocyanin content was noted following the mutation of *MYBL2* (Khusnutdinov et al., 2022). Recent studies have demonstrated that the *APETALA2*-*MYBL2* module inhibits the biosynthesis of proanthocyanidins by influencing the formation of the MBW complex in Arabidopsis seeds (Jiang et al., 2024). Additionally, the negative feedback regulation module, which comprises the R3-MYB repressor *MYBL2* and the R2R3-MYB activator *PAP1*, exerts a fine-tuning effect on anthocyanin biosynthesis induced by high light in Arabidopsis (Xing et al., 2024). However, as the systematic identification and functional analysis of *MYBL2* have not yet been reported, its role in the regulation of anthocyanin synthesis requires further investigation, particularly in *Brassica* crops.

In this study, we systematically identified *MYBL2* homologous genes in 31 cruciferous species with available reference genome information. Excluding *Aethionema arabicum*, we identified a total of 48 *MYBL2* homologous genes across 30 species, and phylogenetic analysis revealed that these genes can be categorized into six subfamilies. Ka/Ks analysis showed that except for 10 genes that were under positive selection during the evolution process, the other genes were under purifying selection. We subsequently analyzed the evolution and collinearity of 16 *MYBL2* homologous genes in six *Brassica* crops, which were found to be divided into two branches, demonstrating significant collinearity on the A, B, and C subgenome chromosomes. Furthermore, our analysis of *cis*-acting elements, gene structure, conserved domains, and conserved motifs in the promoter regions indicated that *MYBL2* is highly conserved throughout evolution, with the exception of *BjuMYBL2.A02* in *B. juncea*, which exhibited a partial deletion in the R3 domain. Additionally, we utilized RNA-seq data to investigate the expression patterns of *MYBL2* in *Brassica*, revealing that expression levels were significantly up-regulated in tissues where anthocyanins accumulate. We also conducted WGCNA analysis in conjunction with RNA-seq data from various tissues of *B. napus*, identifying *BnaMYBL2.C06* as a potential hub gene regulating flower color formation in *B. napus*. The results from qRT-PCR corroborated that the expression patterns of four *MYBL2* genes in *B. napus* aligned with those of ABGs, showing significant up-regulation in leaves and petals. Our findings provide new insights into the role of *MYBL2* in anthocyanin biosynthesis.

## Materials and methods

### Identification, phylogenetic and syntenic analysis of *MYBL2* gene in Brassicaceae

BLASTN and BLASTP alignments (coverage = 60%, identity = 60%, e-value = 1.00e-20) were performed on 31 species of Brassicaceae, using the *MYBL2* (*AT1G71030*) nucleic acid sequence and the protein sequence of Arabidopsis thaliana as references to obtain homologous sequences. The protein sequence of the homologous gene was extracted and submitted to BatchCDD (https://www.ncbi.nlm.nih.gov/Structure/bwrpsb/bwrpsb.cgi) for conservative domain analysis, and the results were compared with those from BLAST to determine the gene family members. The IQ-TREE software was employed to construct the phylogenetic tree with maximum likelihood, while iTOL (https://itol.embl.de) was utilized to enhance the visual representation of the phylogenetic tree. In six *Brassica* species, SynOrthos software was used to analyze the collinear relationships between *A. thaliana* and *Brassica* species (Cheng et al., 2012), and TBtools-II software (v2.118) facilitated the visual analysis (Chen et al., 2023). The coding sequences (CDS) and protein sequences of *MYBL2* family members were retrieved from 30 Brassicaceae genomes, and the Ka/Ks values for each *MYBL2* family member were computed using TBtools-II software (v2.118) (Chen et al., 2023). The R software package pheatmap was used to create the heat map for *MYBL2* members with Ka/Ks values.

### Structure and evolution analysis of *MYBL2* homologous genes in *Brassica*

MEME (https://meme-suite.org/meme/) was employed to analyze the conserved characteristics of the MYBL2 protein sequence, while BatchCDD (https://www.ncbi.nlm.nih.gov/Structure/bwrpsb/bwrpsb.cgi) was utilized to assess the conserved domains of the MYBL2 protein. The 2 kb sequence upstream of the *MYBL2* start codon was extracted and submitted to PlantCARE (http://bioinformatics.psb.ugent.be/webtools/plantcare/html/) for promoter *cis*-acting element prediction analysis, with visualization performed using TBtools-II (v2.118) software (Chen et al., 2023). The protein sequence of the *Brassica MYBL2* homologous gene was submitted to CLUSTALW (https://www.genome.jp/tools-bin/clustalw) for sequence alignment, and the resulting protein sequence alignments were visualized using ESPript3.0 (https://espript.ibcp.fr/ESPript/cgi-bin/ESPript.cgi) and WebLogo3 (https://weblogo.threeplusone.com), respectively.

### Transcriptome sequencing data analysis

To investigate the expression patterns of the *MYBL2* gene across various species of *Brassica* and different tissues of *B. napus*, we collected transcriptome data from the leaves (green and purple) of *B. rapa*, *B. juncea*, and *B. oleracea*. Additionally, the transcriptome data encompassed five distinct tissues of *B. napus*, which included siliques (green and purple), stems (green and purple), leaves (green and purple), flower colors (white and purple), seed coats (yellow and black), as well as six different flower colors (white, beige, yellow, purple, apricot pink, and orange). Furthermore, we obtained transcriptome data from four different developmental stages (S1, S2, S3, S4) of purple flower *B. napus*. Each group of RNA-seq data above contained three biological replicates. The raw RNA-seq data obtained were mapped to the pak choi reference genome (Thakur, 2024) using HISAT2 software (v2.1.0), ensuring that each read matched only one region. The htseq-count function of the HTSeq software (v0.11.2) package was employed to count the number of reads aligned to each gene (Anders et al., 2015). Subsequently, the DEseq2 package (http://www.bioconductor.org/packages/release/bioc/html/DESeq2.html) was utilized to analyze differentially expressed genes (DEGs). The expression level of each gene was calculated using StringTie software (v2.1.1) (Thakur, 2024). Additionally, TBtools-II software (v2.118) was used to create a heat map of gene expression related to the anthocyanin synthesis pathway (Chen et al., 2023).

### WGCNA analysis

To further investigate the hub genes involved in regulating anthocyanin synthesis in the *B. napus* different tissues, we employed WGCNA to construct the interaction relationships among ABGs across leaves (green and purple), stems (green and purple) and flowers (white and purple) of *B. napus*. Correlation analysis was conducted using the WGCNA software package (v4.3.1) (Langfelder and Horvath, 2008) to assess the relationships between each co-expression module, phenotypic traits, and anthocyanin content, thereby identifying key hub genes that influence anthocyanin synthesis. Subsequently, the gene regulatory network data derived from the WGCNA analysis were imported into Cytoscape (v3.80) for visual representation (Shannon et al., 2003). For detailed methodology, please refer to the article by Liu et al (Liu et al., 2024).

### Quantitative analysis

The leaves (green and purple), stems (green and purple) and flowers (white and purple) were collected and then used to extract total RNA, from which cDNA was synthesized through reverse transcription for quantitative real-time PCR (qRT-PCR) analysis. The specific methodology is as follows: First, the Eastep^®^ Super Total RNA Extraction Kit from Promega Biotech Co., Ltd (Beijing, China) was utilized for total RNA extraction. Subsequently, the HiScript III 1st Strand cDNA Synthesis Kit (+gDNA wiper) from Vazyme Biotech Co., Ltd (Nanjing, China) was employed for cDNA synthesis via reverse transcription. The qRT-PCR was conducted using the SYBR qPCR Master Mix from Vazyme Biotech Co., Ltd (Nanjing, China). The reaction mixture comprised a total volume of 20.0 µL, consisting of 10.0 µL SYBR qPCR Master Mix, 0.5 µL each of forward and reverse primers, 4.0 µL of cDNA template, and 5.0 µL of ddH_2_O. The amplification procedure included an initial denaturation step at 95 °C for 30 seconds, followed by 40 cycles of denaturation at 95 °C for 10 seconds and annealing/extension at 60 °C for 30 seconds. This was followed by a final denaturation at 95 °C for 15 seconds, annealing at 60 °C for 60 seconds, and a melting curve analysis at 95 °C for 15 seconds. The relative expression levels were calculated using the 2 ^-ΔΔCT^ method, with the fold change determined based on these relative expression levels. The average values were visualized using Prism 9 (v9.5.1) software. Primer information for the qRT-PCR analysis is provided in **Supplementary Table S1**. For detailed methods, please refer to Chen et al (Chen, 2020).

## Results

### Identification and phylogenetic analysis of *MYBL2* in Brassicaceae species

To systematically identify *MYBL2* genes in Brassicaceae plants, we utilized the *MYBL2* protein sequence of *A. thaliana* (*AT1G71030.1*) as a reference. This approach enabled the identification of *MYBL2* genes across 31 Brassicaceae plants with published reference genomes. The results revealed the presence of *48 MYBL2* genes, excluding *Aethionema arabicum* (**Table 1**; **Supplementary Table S2**). Among these, *B. napus* exhibited the highest number of homologous *MYBL2* copies, totaling four. Additionally, three copies were found in *B. carinata*, *B. juncea*, *Raphanus sativus*, and *Lepidium meyenii*. Two homologous *MYBL2* copies were identified in *B. nigra*, *B. oleracea*, *B. rapa*, *Camelina sativa*, *Leavenworthia alabamica*, *Orychophragmus violaceus*, and *Sinapis arvensis*, while only one *MYBL2* homologous gene was detected in the remaining species (**Table 1**).

**Table 1:** Genome version information and *MYBL2* homologous genes in 31 Brassicaceae species.

Species	Version	Blast	CDD	Identified
*Arabidopsis_thaliana*	Tair10	1	1	1
*Arabidopsis_halleri*	A.halleri-v2.2	1	1	1
*Arabidopsis_lyrata*	Lyrate-v2.1	1	1	1
*Arabis alpina*	Gray-v4.0	1	1	1
*Aethionema arabicum*	A.arabicum-v1.0	0	0	0
*Barbarea vulgaris*	Bittercress-v1.0	1	1	1
*Boechera retrofracta*	Holboell-v1.0	1	1	1
*Boechera stricta*	Drummond-v1.2	1	1	1
*Brassica carinata*	Zd-1-v1.0	3	3	3
*Brassica juncea*	SCYZ	3	3	3
*Brassica napus*	Darmor-v10.0	4	4	4
*Brassica nigra*	Ni100_LR-v2.0	2	2	2
*Brassica oleracea*	JZS-v2.0	2	2	2
*Brassica rapa*	Chiifu-v3.5	2	2	2
*Camelina sativa*	Camelina-v2.0	2	2	2
*Capsella grandiflora*	C.grandiflora.v1.0	1	1	1
*Capsella rubella*	Red_shepherd-v1.1	1	1	1
*Cardamine hirsuta*	Hairy-v1.0	1	1	1
*Eutrema salsugineum*	173-v1.0	1	1	1
*Isatis indigotica*	Woad-v1.0	1	1	1
*Leavenworthia alabamica*	Alabama-v1.0	2	2	2
*Lepidium meyenii*	Peruvian-v1.0	3	3	3
*Matthiola incana*	M.incana-v1.0	1	1	1
*Microthlaspi erraticum*	M.erraticum-v1.0	1	1	1
*Orychophragmus violaceus*	O.violaceus-v1.0	2	2	2
*Raphanus sativus*	Radish-v1.0	3	3	3
*Schrenkiella parvula*	Saltwater-v1.0	1	1	1
*Sinapis alba*	S.alba-v1.0	1	1	1
*Sinapis arvensis*	S.arvensis-v1.0	2	2	2
*Sisymbrium irio*	London-v1.0	1	1	1
*Thlaspi arvense*	Field-v1.1	1	1	1
Total	31	48	48	48

**Figure 1:**
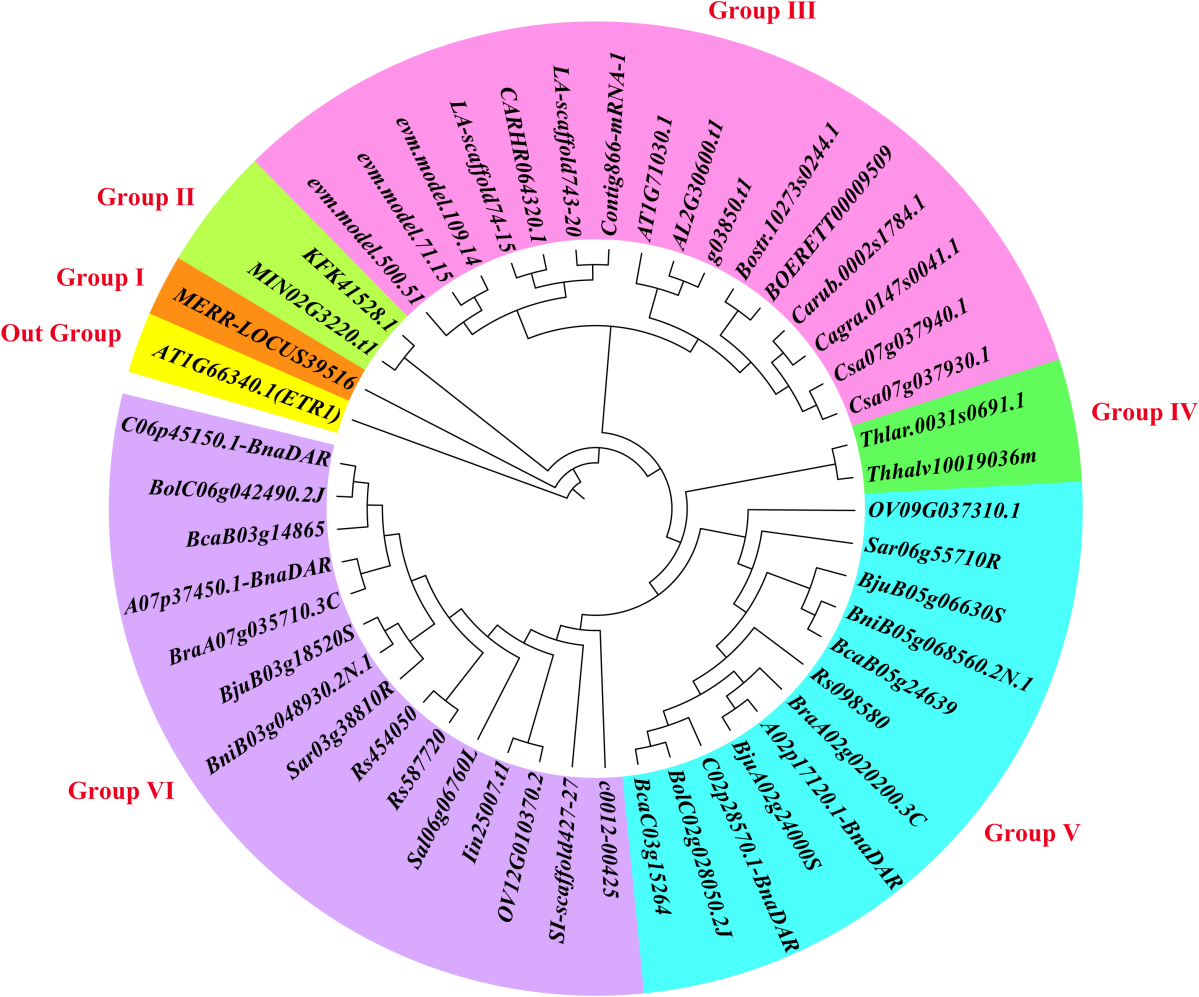
Phylogenetic relationships of *MYBL2* homologous genes between 31 Brassicaceae species. The 48 homologous genes of *MYBL2* were divided into six subfamilies, named Group I to Group VI by different colors, and AT1G66340.1 was divided into a separate group as an out group.

To investigate the evolutionary relationships among *MYBL2* homologous genes across 31 species of Brassicaceae, we constructed a phylogenetic tree using protein sequences. The phylogenetic analysis revealed that 48 *MYBL2* homologous genes can be classified into six subfamilies (Group I to Group VI) (**Figure 1**). Notably, the *rna-MERR-LOCUS39516* from *Microthlaspi erraticum* forms a separate group (Group I), while Group II comprises two genes: *MIN02G3220.t1* from *Matthiola incana* and *KFK41528.1* from *Arabis alpina*. Group III includes 16 members of the *MYBL2* gene family, among which *AT1G71030.1* is represented. Additionally, *Thlar.0031s0691.1* from *Thlaspi arvense* and *Thhalv10019036m* from *Eutrema salsugineum* are classified within Group IV. Groups V and VI contain 12 and 15 genes, respectively, with 16 *MYBL2* homologous genes from six species of *Brassica* distributed across these two groups. Ka/Ks analysis found that only 20 gene pairs had Ka/Ks values > 1, indicating that they were subject to positive selection, while the Ka/Ks values of other gene pairs were < 1, indicating that these genomes were mainly subject to purifying selection during evolution (**Supplementary Figure S1**; **Supplementary Table S3**).

### Evolution and collinearity analysis of *MYBL2* homologous genes in six species in U’s triangle

**Figure 2:**
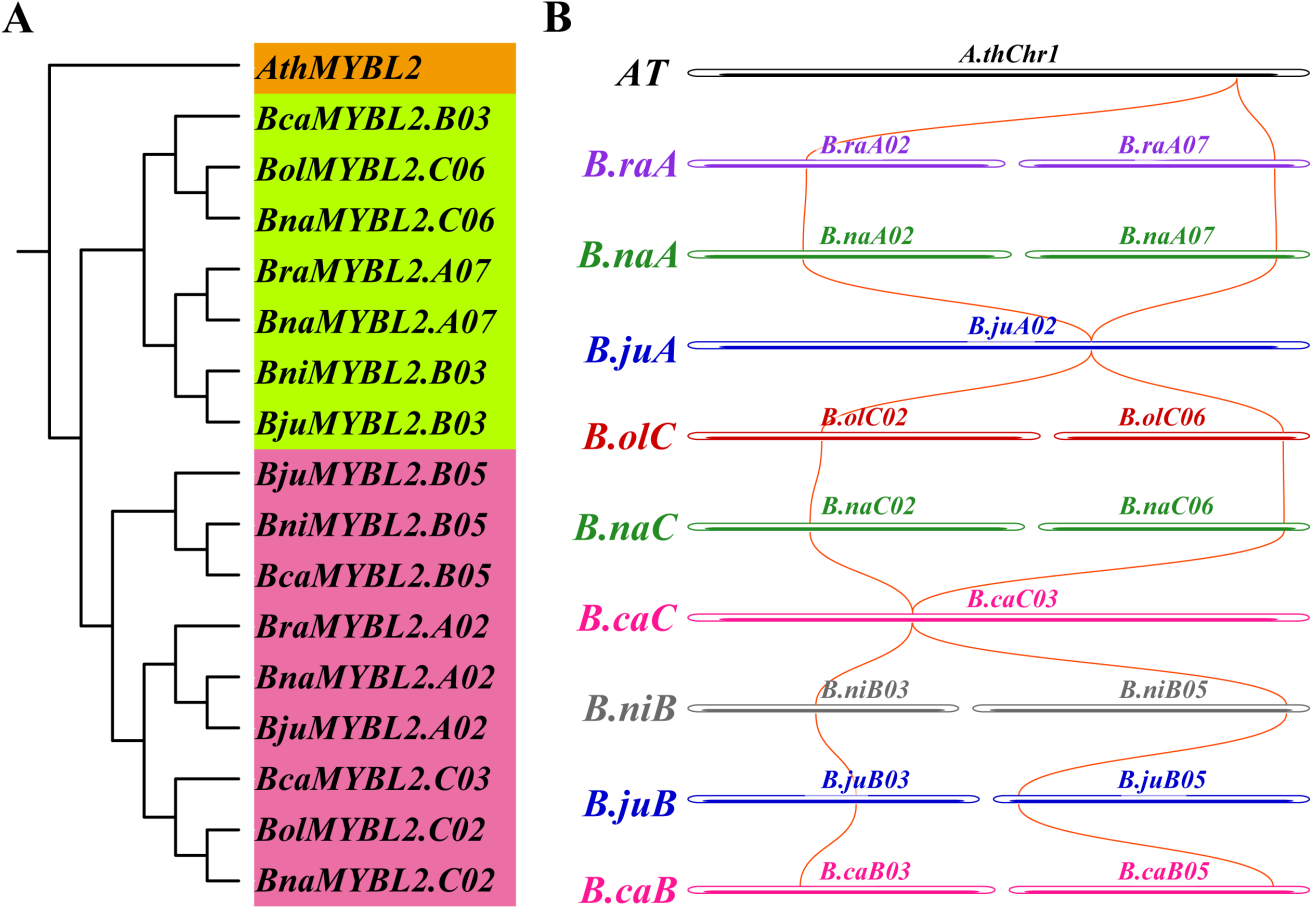
Evolution and collinearity analysis of 16 *MYBL2* homologous genes in *Brassica* species. **(A)** Phylogenetic tree of 16 *MYBL2* homologous genes; **(B)** Colinearity of 16 *MYBL2* homologous genes among A, B and C subgenomes.

In six *Brassica* species of U’s triangle, a total of 16 *MYBL2* homologous genes were identified (**Table 1**). To clarify the evolutionary relationships among 16 *MYBL2* homologous genes in six *Brassica* species, we conducted phylogenetic and collinearity analyses of these genes (**Figure 2**; **Supplementary Table S4**). The results indicated that the 16 *MYBL2* homologous genes were categorized into two primary branches, which could further be subdivided into three smaller branches corresponding to the three subgenomes: A, B, and C (**Figure 2A**; **Supplementary Table S5**). Notably, within these three subgenomes, the *MYBL2* homologous genes located on chromosomes A07, C06, and B03 clustered within the same evolutionary branch. In contrast, the *MYBL2* homologous genes found on chromosomes A02, C02, C03, and B05 were allocated to a different evolutionary branch, suggesting that the homologous genes in these two branches may have diverged early in their evolutionary history. Furthermore, we examined the co-linearity of these 16 *MYBL2* homologous genes across the *Brassica* A, B, and C subgenomes. The findings revealed that in the A subgenome, the *MYBL2* homologous genes were predominantly located on the A02 and A07 chromosomes of *B. napus* and *B. rapa*, while *B. juncea* contained only one homologous gene on the A02 chromosome. In the C subgenome, *MYBL2* homologous genes were mainly found on chromosomes C02 and C06 of *B. napus* and *B. oleracea*, with only one homologous gene from *B. carinata* located on chromosome C03. In the B subgenome, *MYBL2* homologous genes were primarily distributed across the B03 and B05 chromosomes of *B. nigra*, *B. juncea*, and *B. carinata* (**Figure 2B**; **Supplementary Table S6**).

### Structure analysis of 16 *MYBL2* homologous genes in six *Brassica* species

**Figure 3:**
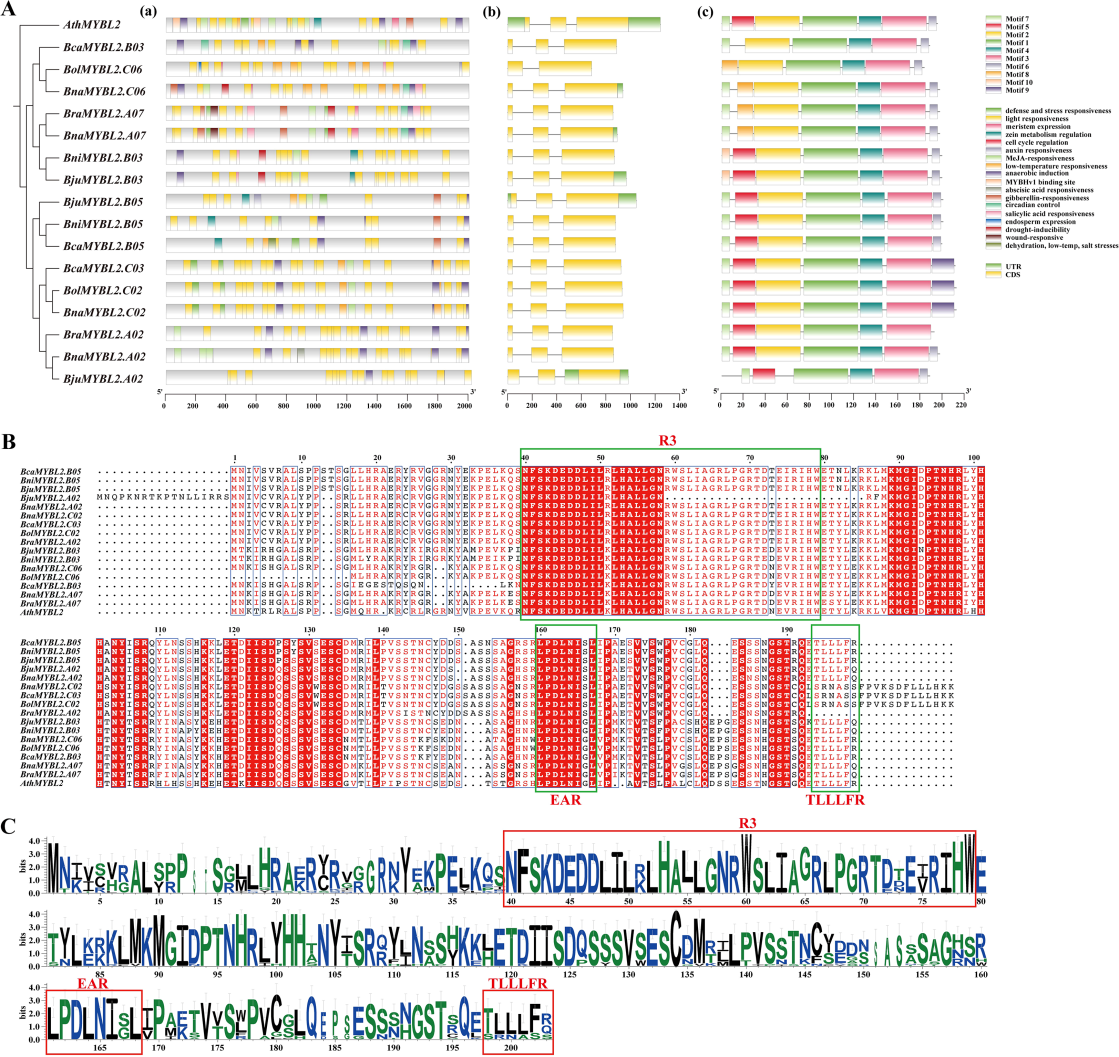
Analysis of *cis*-acting elements, gene structure, conserved motifs, conserved domains and protein sequence alignment of *MYBL2* homologous gene promoters in six *Brassica* species. **(A)** Promoter *cis*-acting elements, gene structure, conserved motif and conserved domain analysis composite diagram, (a) promoter *cis*-acting elements, (b) gene structure, (c) conserved motif; **(B)** Conserved domain analysis of *MYBL2* in six *Brassica* species, in the green box are R3 domain, EAR domain and TLLLF domain, respectively; **(C)** R3 conservative motif analysis *MYBL2* in six *Brassica* species, in the red box are R3 motif, EAR motif and TLLLF motif.

To comprehensively study the evolutionary patterns of 16 *MYBL2* homologous genes in *Brassica*, we analyzed the promoter *cis*-acting elements, gene structure, conserved motifs, and conserved domains of these genes (**Figure 3**). Initially, we predicted the *cis* -acting elements within the 2 Kbp promoter sequence upstream of the 16 *MYBL2* homologous genes, identifying a total of 39 distinct *cis*-acting elements (**Supplementary Table S7**). These elements were classified into categories including anaerobic induction, auxin responsiveness, cell cycle regulation, circadian control, defense and stress responsiveness, dehydration, low-temperature and salt stresses, low-temperature responsiveness, endosperm expression, gibberellin responsiveness, abscisic acid responsiveness, light responsiveness, MeJA responsiveness, meristem expression, drought inducibility, MYBHv1 binding site, salicylic acid responsiveness, wound responsiveness, and zein metabolism regulation, totaling 18 types of *cis*-regulatory elements. Notably, *cis* -acting elements associated with light response constituted half of the total. The distribution of components within the same branch exhibited similarities (**Figure 3A**(a)). The gene structure of the 16 *MYBL2* homologous genes was highly conserved; with the exception of the deletion of the first exon in the gene *Bol.MYBL2.C06*, the remaining 15 homologous genes comprised three exons, and the total gene length, as well as the length and location of each exon, were highly similar (**Figure 3A**(b)). MEME online software was utilized to analyze 16 homologous MYBL2 proteins motifs, resulting in the identification of a total of 10 distinct motifs. The analysis revealed that all homologous genes contained motifs 1, 3, and 4; however, the gene *BjuMYBL2.A02* uniquely lost motif 2 and exhibited a partial deletion at the N-terminus (**Figure 3A**(c); **Supplementary Table S8**).

Interestingly, both the *cis*-acting elements of the promoter and the gene structure, including conserved motifs and domains, reveal that the gene *BjuMYBL2.A02* is significantly different from other members of the *Brassica* family. To further investigate this, we compared the protein sequences of these 16 homologous genes. The results indicated that the gene *BjuMYBL2.A02* lacks a complete R3-MYB structure (**Figure 3B**), while the remaining 15 homologous genes possess a complete R3-MYB structure. Additionally, motif analysis of the 16 *MYBL2* protein sequences in *Brassica* demonstrated that they exhibit a typical R3 conserved motif, EAR motif (LPDLNI(S/G)L) and a terminal TLLLF motif (**Figure 3C**). These findings suggest that, with the exception of *BjuMYBL2.A02*, *MYBL2* is highly conserved across *Brassica* species.

### Expression pattern analysis of *MYBL2* homologous genes in *Brassica* species

**Figure 4:**
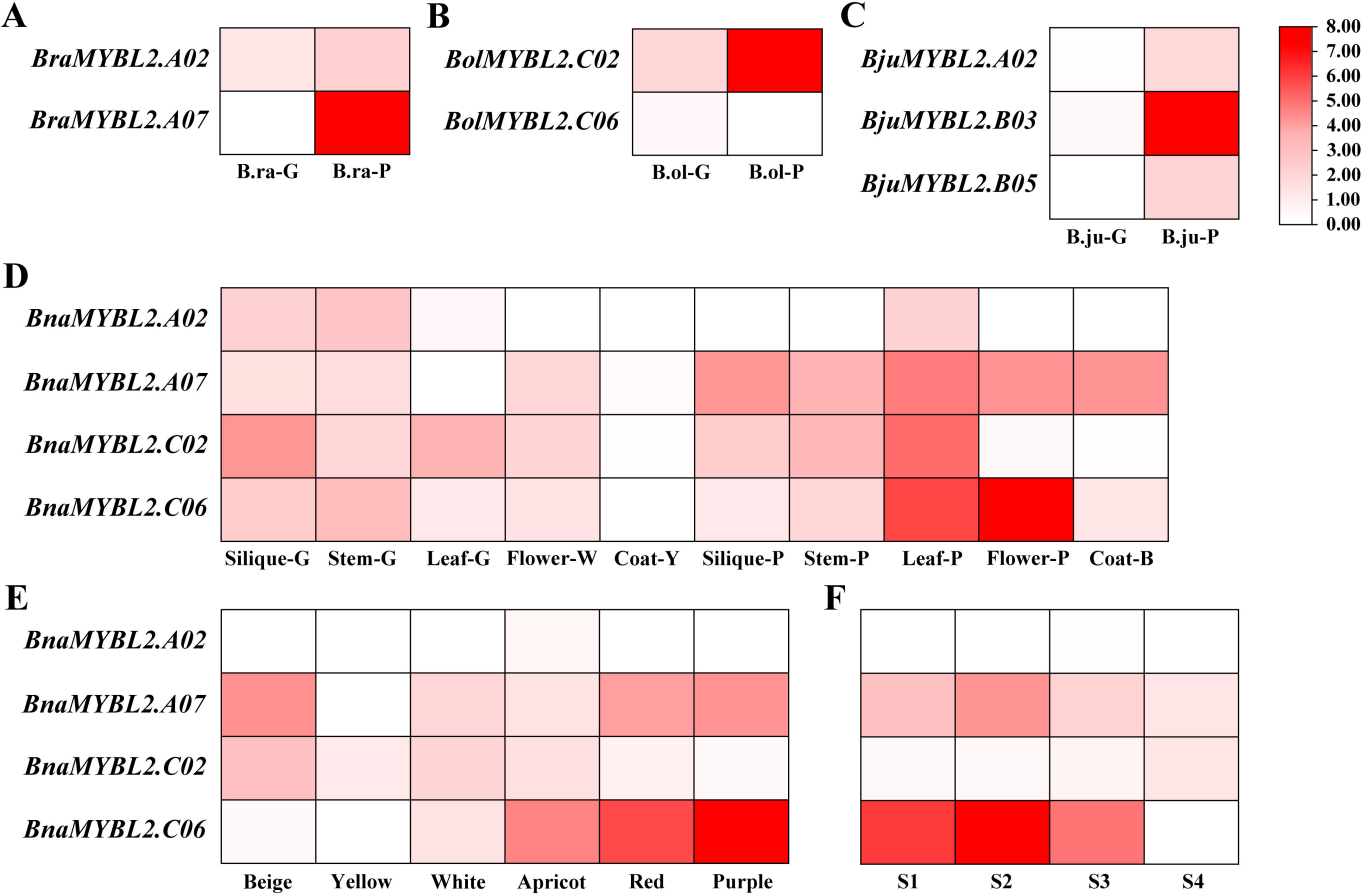
Gene expression heat map of *MYBL2* homologous genes in *Brassica* species. **(A)** Green and purple leaf of *B*. *rapa*; **(B)** Green and purple leaf of *B*. *oleracea*; **(C)** Green and purple leaf of *B*. *juncea*; **(D)** Different tissues of *B*. *napus*; **(E)** Different flower colors of *B*. *napus*; **(F)** Different developmental stages of *B*. *napu* purple petals.

To investigate the role of *MYBL2* homologous genes in anthocyanin biosynthesis within *Brassica* crops, we utilized existing RNA-seq data to analyze the expression patterns of these genes in the leaves (purple and green) of *B. rapa*, *B. oleracea*, and *B. juncea*, as well as in various tissues of *B. napus*, including different flower colors and purple petals at various developmental stages (**Figure 4**; **Supplementary Table S9**). The results indicated that *BraMYBL2.A02* was significantly up-regulated in the purple leaves of *B. rapa* (**Figure 4A**), *BolMYBL2.C06* was significantly up-regulated in the purple leaves of *B. oleracea* (**Figure 4B**), and *BjuMYBL2.B03* was significantly up-regulated in the purple leaves of *B. juncea*. Additionally, the expression levels of *BjuMYBL2.A02* and *BjuMYBL2.B05* in purple leaves were also found to be higher than those in green leaves (**Figure 4C**).

In *B. napus*, we first examined the expression patterns of four *MYBL2* homologous genes across different tissues: siliques (green and purple), leaves (green and purple), stems (green and purple), petals (white and purple), and seed coats (yellow and black). Our findings revealed that the expression levels of these four *MYBL2* homologous genes were higher in purple leaves compared to green leaves, with *BnaMYBL2.A07* showing significant up-regulation in tissues exhibiting anthocyanin accumulation (**Figure 4D**). Moreover, *BnaMYBL2.A07* and *BnaMYBL2.C06* were highly expressed in petals of various colors, particularly in those with anthocyanin accumulation (**Figure 4E**; **Supplementary Figure S2**). Furthermore, we analyzed the expression patterns of the four *MYBL2* homologous genes during the four developmental stages of purple petals. We found that *BnaMYBL2.C06* was significantly highly expressed in stages S1 to S3, while *BnaMYBL2.A07* exhibited high expression across all stages (S1 to S4), with the highest expression level observed in stage S2 (**Figure 4F**).

### Co−expression network analysis and identification of hub genes for anthocyanin biosynthesis regulation

**Figure 5:**
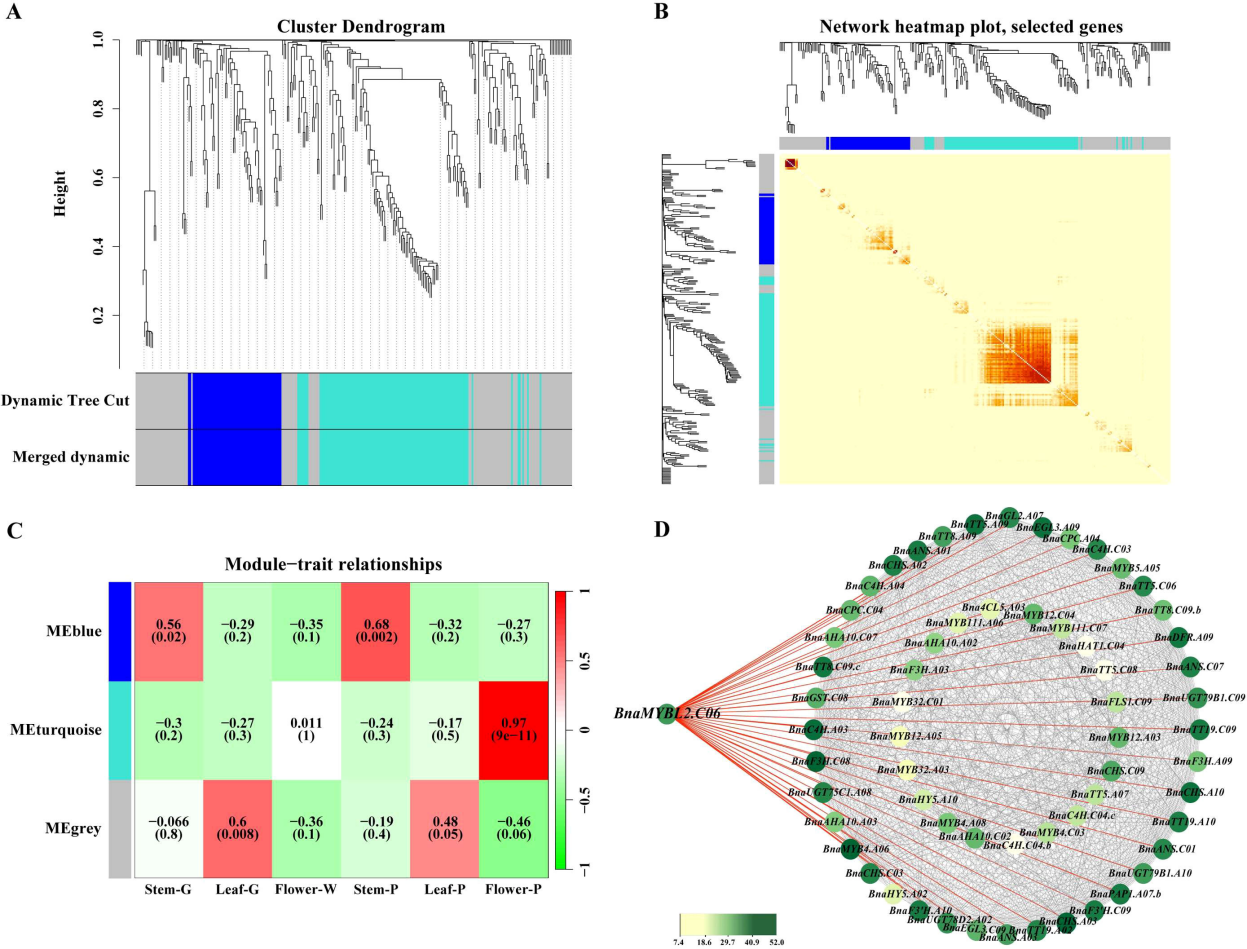
Weighted gene co-expression network analysis of ABGs in different tissues of *B*. *napus*. **(A)** The cluster dendrogram tree showing three modules of co-expressed genes by WGCNA, each leaf of tree corresponds to one gene, and the tree branches constitute three modules, labeled with different colors; **(B)** The heat map results of module clustering and the relationship, a clustering algorithm is employed to group various co-expression modules, thereby illustrating the correlations among them, the correlation between modules is further depicted in the module correlation matrix below, both the horizontal and vertical axes represent gene modules, and a deeper red color indicates a higher correlation, while a greener color signifies a lower correlation between the modules; **(C)** Module-to-sample correlation heatmap, correlation analysis was performed between the co-expression modules of various genes associated with ABGs in different tissues, the numbers above the heat map indicate the Pearson correlation coeffcient (r) values; **(D)** Cytoscape representation of co-expressed anthocyanin metabolism related genes w in the MEturquoise module.

To further investigate the role of *MYBL2* in the regulation of anthocyanin synthesis across various tissues of *B. napus* and to identify hub genes, we selected leaves (green and purple), stems (green and purple), and petals (white and purple) from *B. napus*. Six sample groups, each consisting of three biological replicates, were subjected to RNA sequencing, and the resulting phenotypic data were analyzed using WGCNA (**Figure 5**).The results indicated that these genes were primarily clustered into three modules: MEblue, MEturquoise, and MEgrey (**Figure 5A**), with the MEblue module exhibiting the strongest gene interactions (**Figure 5B**). Subsequently, we conducted a correlation analysis among the three modules across the six sample groups, revealing that the MEturquoise module displayed the strongest correlation in purple petals (**Figure 5C**). Furthermore, we examined the regulatory relationships of ABGs within this module, identifying 62 enriched genes and complex regulatory interactions among them (**Figure 5D**). Notably, among the four *MYBL2* homologous genes in *B. napus*, only *BnaMYBL2.C06* was present in this module, and it exhibited regulatory relationships with 40 ABGs, further supporting the hypothesis that *BnaMYBL2.C06* may function as a hub gene regulating anthocyanin synthesis.

### Expression analysis of anthocyanin biosynthesis genes in different tissues of *B. napus* by qRT-PCR

**Figure 6:**
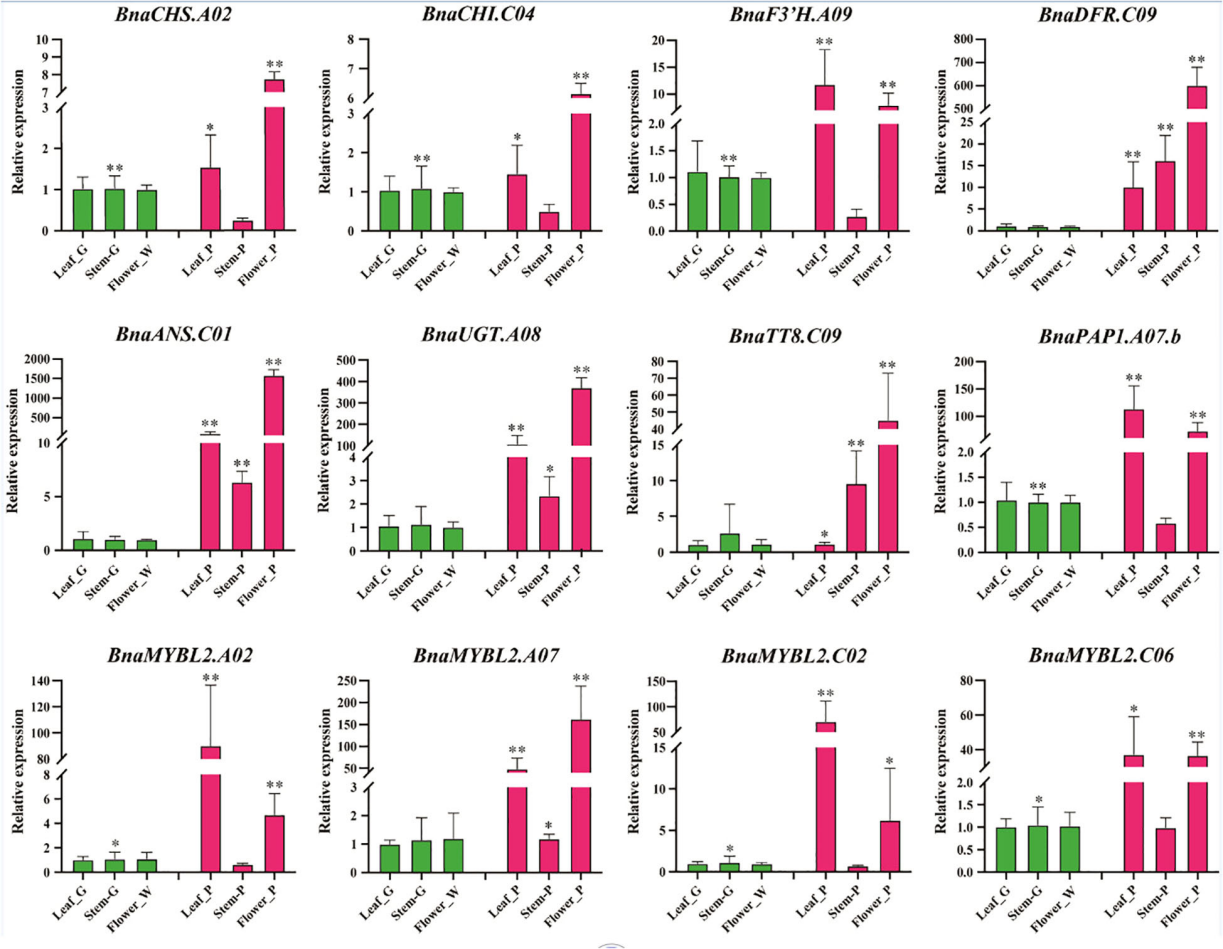
qRT-PCR analysis of expression patterns of anthocyanin biosynthesis genes in different tissues of *B*. *napus*. For qRT-PCR analysis, both white and purple flower samples are with three biological replicates, the point represents the mean value of three technical replicates in a representative biological experiment, the error bars indicate s.d, student’s t-test, **P<0.01, *P<0.05.

To further validate the results of the RNA-seq analysis, we conducted qRT-PCR validation using leaves (green and purple), stems (green and purple), and petals (white and purple) of *B. napus*. We selected eight genes related to the anthocyanin biosynthetic pathway (*BnaCHS.A02*, *BnaCHI.C04*, *BnaF3H.A09*, *BnaDFR.C09*, *BnaANS.C01*, *BnaUGT.A08*, *BnaTT8.C09*, *BnaPAP1.A07.b*) and four *MYBL2* homologous genes in *B.* napus for qRT-PCR analysis (**Figure 6**). The results indicated that eight ABGs were significantly differentially expressed in purple leaves and petals, particularly the key genes *BnaDFR.C09* and *BnaANS.C01*, which catalyze the conversion of flavonoids to anthocyanins, showing significant up-regulation in all three purple tissues. Notably, four homologous copies of *MYBL2* exhibited significant differential expression in purple petals and purple leaves, but not in the purple and green stems. This expression pattern was analogous to that of the genes *BnaCHS.A02*, *BnaCHI.C04*, *BnaF3H.A09*, *BnaANS.C01*, and *BnaUGT.A08*. Importantly, the expression pattern of the four *MYBL2* homologs aligns with that of the known positive regulator of anthocyanin biosynthesis, *BnaPAP1.A07.b*.

## Discussion

### Evolution of *MYBL2* in Brassicaceae species

*MYBL2* is a typical R3-MYB transcription regulator, belonging to the S4-derived R3-MYB subclass, characterized by a partial R2 domain, an EAR-repression motif, and a TLLLFR inhibitory domain (Khusnutdinov et al., 2021; LaFountain and Yuan, 2021). In this study, we systematically identified *MYBL2* homologous genes in 31 Brsssicaceae species with published reference genome information, based on the *AtMYBL2* sequence of *A. thaliana*, resulting in a total of 48 identified genes. Notably, no *MYBL2* homologous gene was found in *A. arabicum*, which may be attributed to the loss of the *MYBL2* gene during evolution or to incomplete genome assembly. Interestingly, the copy number of *MYBL2* homologous genes within the same species closely aligns with the phylogeny and evolution of the Brassicaceae family (Murat et al., 2015; Oh and Dassanayake, 2019). For instance, there is only one *MYBL2* homologous gene in *A. lyrata*, *A. haleri*, and other plants that have not undergone genome-wide doubling events, whereas there are two *MYBL2* homologous genes in *B. rapa*, *B. oleracea*, and other species that have experienced such doubling events. Allotetraploid species, such as *B. juncea* and *B. carinata*, possess three *MYBL2* homologous genes, while *B. napus* has four. The Ka/Ks analysis results of 48 *MYBL2* homologous genes showed that only 10 genes were positively selected during the evolution process, these genes were mainly concentrated in species with more human selection, such as *Brassica*, while the other *MYBL2* homologous genes were mainly purifying selected during the evolution process. Furthermore, phylogenetic and collinearity analyses of 16 *MYBL2* homologous genes in *Brassica* crops provide further evidence supporting this observation, indicating that the distribution of these 16 *MYBL2* homologous genes across the A, B, and C subgenomes originated from a common ancestor but differentiated at an earlier stage. The distribution of these genes on different chromosomes of the A, B, and C subgenomes reflects the orthologous relationship of these two genes. At the same time, conserved domain analysis found that the *BjuMYBL2.A02* gene exhibits a deletion of the R3 domain, which indicates that the gene structure of *MYBL2* has also changed during species evolution. However, the genomes of the allotetraploids *B. juncea*, *B. carinata*, and *B. napus* are currently at the telomere-to-telomere level due to the presence of high repetitive sequences, necessitating further investigation as subsequent genomic information becomes available.

### *MYBL2* regulates plant anthocyanin synthesis

The regulation of *MYBL2* on anthocyanin biosynthesis in plantswas first reported in *A. thaliana*. It was discovered that *MYBL2* work as a repressor that competes with *PAP1/2* for binding to *GL3/TT8*, thereby inhibiting the formation of the *PAP1/2*-*GL3/TT8*-*TTG1* transcriptional regulatory complex, which results in the negative regulation of anthocyanin biosynthesis in *A. thaliana* (Dubos et al., 2008; Matsui et al., 2008). Knockout of *AtMYBL2* in *A. thaliana*, the expression levels of the key gene *DFR* and the regulatory gene *TT8* were found to be up-regulated, resulting in a significant increase in anthocyanin content (Dubos et al., 2008). In addition, *HY5* can inhibit *MYBL2* activity by activating the expression of *MYBD*, which in turn increases anthocyanin accumulation in *A. thaliana* (Nguyen et al., 2015). And it has been observed that *MYBL2* can interact with *AP2* to form the *AP2-MYBL2-TT8/EGL3* complex, which disrupts the formation of the MBW complex. This disruption inhibits the expression of *ANR*, *TT12*, *TT19*, and *AHA*, thereby hindering the biosynthesis of proanthocyanidins in *A. thaliana* seeds (Jiang, ). In *Brassica* crops, *BrMYBL2.1* is considered to negatively regulate anthocyanin biosynthesis in Zicaitai (*B. rapa*) and Chinese cabbage (Zhang et al., 2020; Kim et al., 2022), while the anthocyanin content in *B. oleracea* increased following *MYBL2* mutation (Khusnutdinov et al., 2022). These findings suggest that the function of *MYBL2* in *B. rapa* and *B. oleracea* is consistent with that in *A. thaliana*, where it negatively regulates anthocyanin biosynthesis. However, in this study, we observed that the expression patterns of four *MYBL2* homologous genes in the leaves, stems, and petals of *B. napus* aligned with the trend of ABGs, with expression levels significantly up-regulated in tissues exhibiting high anthocyanin content. This indicates a close relationship between *MYBL2* and anthocyanin biosynthesis in *B. napus*. Moreover, we found that the expression pattern of *MYBL2* was consistent with that of *BnaPAP2.A7b*, and their competing bHLH transcription factor *TT8*, along with the regulated structural gene *DFR*, also exhibited similar patterns. These results further suggest that as plant anthocyanins are synthesized and accumulated, the expression of *MYBL2* is up-regulated, competing with *BnaPAP2.A7b* for *TT8* to inhibit further anthocyanin synthesis. Recent studies have revealed that *MYBL2* interacts with *PAP1/2* to diminish its transcriptional activation activity, consequently reducing the expression of key genes *DFR* and *TT19* involved in anthocyanin biosynthesis (Xing et al., 2024). This finding is particularly insightful for our subsequent research, as it elucidates the similar expression trends of *MYBL2* and *PAP1/2* observed in this study. We speculate that the substantial synthesis of anthocyanins triggers a feedback regulation mechanism in *B.napus*. This feedback may disrupt the formation of the MBW complex by enhancing *MYBL2* expression and its interaction with *PAP1/2*, thereby inhibiting further synthesis and accumulation of anthocyanins. The underlying mechanisms warrant further investigation.

## Conclusion

In this study, we systematically identified 48 *MYBL2* homologous genes from 30 cruciferous species, and phylogenetic analysis revealed that these genes can be classified into six subfamilies. Subsequently, we selected 16 *MYBL2* homologous genes from six *Brassica* crops within U’s triangle for evolutionary, gene structure, and conserved domain analyses. Our findings indicated that these 16 *MYBL2* homologous genes predominantly clustered into two branches and exhibited a high degree of evolutionary conservation. Further RNA-seq analysis demonstrated that *MYBL2* homologous genes were significantly up-regulated in tissues exhibiting anthocyanin accumulation. Additionally, WGCNA of different tissues in *B. napus* revealed that *BnaMYBL2.C06* had a strong regulatory relationship with ABGs in petals. Through qRT-PCR analysis of *B. napus* leaves, stems, and petals, we observed that four *MYBL2* homologous genes were significantly up-regulated in leaves and petals, with expression patterns consistent with those of ABGs.

## Data Availability

The original contributions presented in the study are included in the article/**Supplementary Material**. Further inquiries can be directed to the corresponding authors.
