# Supplementary material for: Genome-wide identification of MYBL2 in Brassicaceae, with a focus on the expression pattern of regulating anthocyanin synthesis in Brassica crops
Source: Front Plant Sci. 2025 Jul 1;16:1629560. doi: 10.3389/fpls.2025.1629560 (PMC12259598; doi:10.3389/fpls.2025.1629560)
Supplement: Supplementary file 1 [file DataSheet1.docx]

Supplement figures


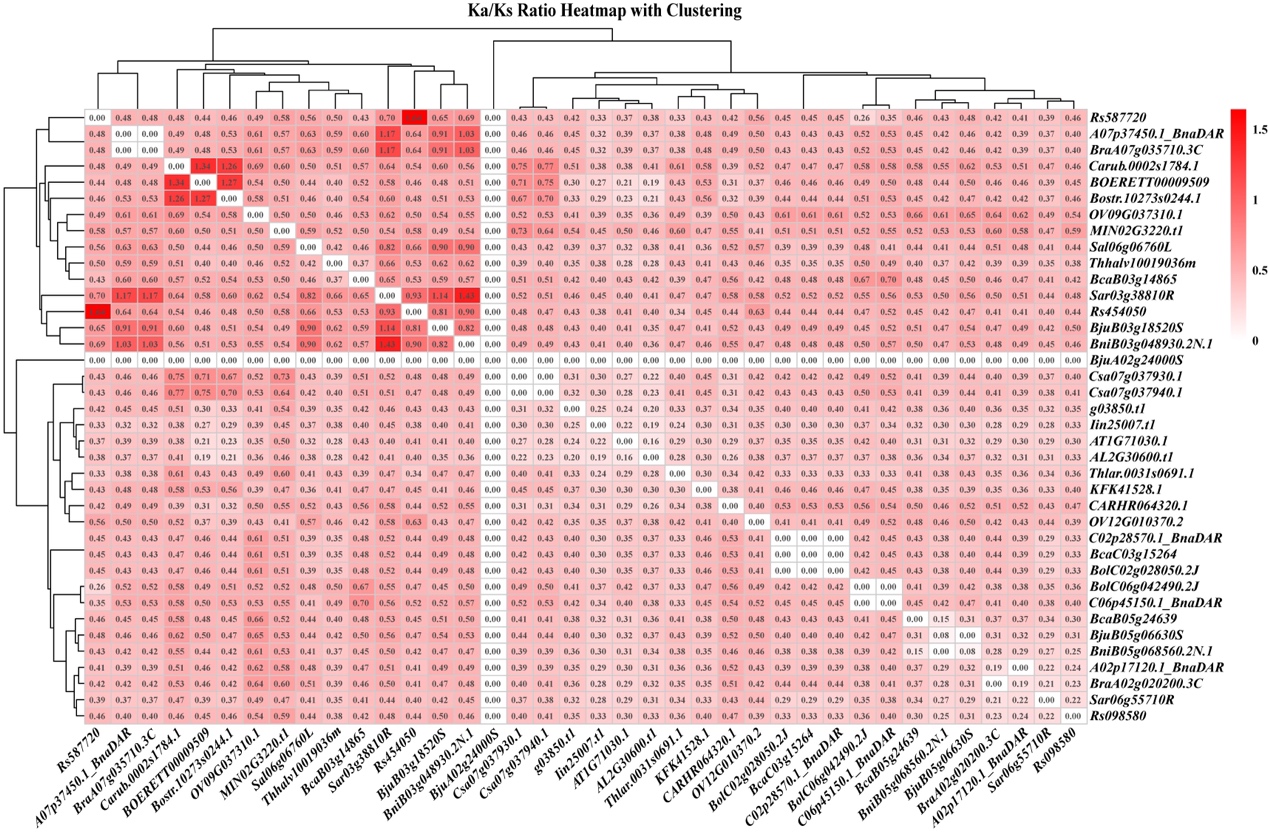


**Figure S1. Ka/Ks analysis of 48 *MYBL2* homologous in Brassicaceae.**

**Figure S2. Expression patterns of anthocyanin-related genes in various flower colors of *B. napus*.** A: Expression patterns of all ABGs across the different flower colors of *B. napus*; B: The expression pattern of the A subgenome; C: Expression patterns of the C subgenome.
